# The spatial effect of digital economy on public psychological resilience during the diffusive crisis

**DOI:** 10.3389/fpubh.2023.1156367

**Published:** 2023-05-18

**Authors:** Jiancong Tao, Zhe Wang, Junwei Li

**Affiliations:** ^1^School of Economics & Management, Beijing Forestry University, Beijing, China; ^2^School of Humanities and Social Sciences, University of Science and Technology of China, Hefei, Anhui, China

**Keywords:** psychological resilience, digital economy, spatial spillover effect, public mental health, diffusive crisis

## Abstract

**Purpose:**

To explore whether the digital economy has spatial effects and spatial heterogeneity on public psychological resilience during the diffusive crisis and to analyze the specific impact mechanisms.

**Methods:**

This study is based on the Baidu Search Index from 2011 to 2020 and the provincial panel data of 30 provinces in China. It constructs measures of public psychological resilience and digital economy development level and employs the spatial Durbin model to empirically analyze the relationship between the two, revealing their spatial impact.

**Results:**

(1) Public psychological resilience exhibits a spatial distribution characterized by high values in the west, medium values in the central region, and low values in the east, while the digital economy development level shows a “U”-shaped spatial structure with high levels in the eastern and western regions and low levels in the middle; (2) The digital economy development level in a local region has a negative effect on the public psychological resilience of that region, while the digital economy development level in surrounding regions has a positive spatial spillover effect on the local region’s public psychological resilience.

**Conclusion:**

It is essential to strengthen crisis management, focus on the coordinated development of the digital economy in different regions, share the benefits of digital society development more equitably and broadly, and further improve the psychological resilience of regions under the context of digital economy development.

## 1. Introduction

Since the outbreak of the COVID-19, the discussion of crisis events has once again drawn intense attention from scholars. Examining the emergence and evolution of crisis events in China in recent years reveals that they are almost always accompanied by the spread and dissemination of non-objective, untruthful information, such as rumors, hearsay, and gossip, or by the distortion and mutation of information. For instance, following the 2011 Fukushima nuclear disaster, panic-buying of salt ensued in China; during the COVID-19 pandemic, many people blindly stockpiled medications without knowing the full story. These chaotic events have severely disrupted normal life and have had a significant impact on the public’s mental health (1). Such crisis that remains diffusive often exhibit rapid transmission, wide dissemination, and unpredictable trends. The occurrence of these events, laden with considerable uncertainties and risks, not only causes immeasurable losses to economic development but also threatens the stability of society and politics. Consequently, effectively preventing and addressing various issues arising during the spread of crisis events has become an important topic of concern for scholars.

When studying negative public events, many researchers investigate the level of public mental health, and psychological resilience is a commonly used index. Psychological resilience, also known as psychological elasticity, refers to a stable psychological characteristic that reduces, adapts to, or even overcomes the negative impact of sudden events on mental health (2). The existence of psychological resilience enables the public to self-regulate and cope with the emotional turmoil incurred by the diffusive crisis, which is crucial for maintaining mental health after such events (3, 4). From the perspectives of safety informatics and information economics, the key to resolving diffusive crisis lies in information management, and the prevention of uncertain risks also depends on the extent of available information. Similarly, the alleviation of negative emotions during the events has been found to depend on the amount of information available to the public, specifically their understanding of the current situation and potential impacts of the crisis. When the public cannot access necessary information, they may experience negative emotions such as panic and anxiety (5). Hence, the psychological resilience of the public following diffusive crisis events is influenced by information dissemination. Therefore, risk management for these events requires strict control over the degree of information dissemination.

In recent years, the development of the digital economy, driven by the Internet and big data, has made information the most fluid element, and various digital media platforms facilitate information dissemination. The transmission of crisis information has broken the unidirectional linear communication model, gradually forming a bidirectional, multi-faceted communication model. This model empowers the public, as information recipients, is no longer passively receiving information but actively seeking additional information and feedback based on their needs (6). Thus, it can be inferred that digital media brought by the digital economy may influence the public’s psychological resilience. A review of previous studies reveals inconsistent conclusions: some scholars argue that, compared to traditional media, the fragmented and complex information disseminated by emerging media cannot guarantee quality, thereby amplifying negative public emotions. In contrast, other scholars contend that despite information overload, there is no significant relationship between the acquisition of information from multiple sources and the intensification of negative emotions (7, 8). Therefore, this study aims to provide empirical evidence on the impact of the digital economy on psychological resilience during diffusive crisis by using quantitative methods and to provide data support for related research.

The First Law of Geography posits that everything in real life has geographical relevance. Existing research has demonstrated that the public’s mental state is influenced by geographical factors during crisis, with the widely recognized and influential “Ripple Effect” being a prime example (9, 10). This theory suggests that during sudden public health events, the psychological state of individuals in different regions may exhibit a “ripple effect,” wherein the closer individuals are to the epicenter of the crisis, the higher their risk perception and negative emotions. The “ripple” is a vivid metaphor for depicting the impact of risk events within the framework of risk society amplification (9). It is analogous to a stone thrown into a calm lake, where the point of impact experiences the greatest disturbance, and the degree of disturbance in the surrounding water decreases as the distance from the center increases. Slovic elaborated on the stone at the center of the ripple, arguing that the severity, mode, and nature of the risk event, as well as the ways in which the public acquires, perceives, and interprets information, all influence the depth and breadth of the ripple effect (10). Subsequent research, based on this concept, has demonstrated that the public’s mental state during specific crisis, such as the COVID-19 pandemic, does not fully conform to the “ripple effect” but rather exhibits a turbulent spatial heterogeneity due to the interaction of information differences and spatial distance (11–14).

In summary, it can be concluded that unlike general public crisis events, the impact of diffusive crisis on public mental health does not entirely align with the concept of the “ripple effect” due to their rapid dissemination and multi-point outbreak trends. However, most existing research is limited to this hypothesis, focusing on a specific region as the event’s epicenter and measuring the spatial characteristics of public mental states in different regions based on the geographical distance between them, neglecting the exploration and analysis of spatial spillover effects among neighboring areas. To address this, the present study utilizes provincial panel data from China between 2011 and 2020, employing time-series global principal component analysis to construct indicator systems for public psychological resilience and digital economic development. Using spatial econometric models, the study investigates the spatial effects and heterogeneity of information media on public psychological resilience under the digital economy. From a geographical perspective, this research aims to contribute to the policy making for the prevention, control, and management of diffusive crisis.

## 2. Mechanism analysis and research hypotheses

The spatial mechanism of the digital economy’s impact on public psychological resilience can be divided into direct effect channels and indirect effect channels. On one hand, during the spread of a diffusive public crisis, the digital economy relies on a large amount of easily accessible information, which narrows the perceived distance between the public and the crisis event, thereby directly affecting public psychological resilience through the ripple effect in psychology. On the other hand, the development of the digital economy may exacerbate regional development imbalances to a certain extent, making the division between groups within and outside regions more pronounced, indirectly affecting public psychological resilience through intergroup threat theory.

### 2.1. Direct effect channels

#### 2.1.1. Enrich information sources and enhance the speed of information dissemination

One of the most important features of the digital economy era is the large amount of information and the rapid dissemination of that information. When a diffusive public crisis occurs, the public can access a large amount of official information and rumors about the crisis on the Internet. Faced with a mix of true and false fragmented information, the public is more likely to accept negative information related to their interests (15). The digital economy, as an accelerator of information, is a double-edged sword. At present, although the internet has some mechanisms to verify information, it still cannot keep up with the speed of spreading false negative information. Therefore, when the level of digital economy development increases, it will broaden the public’s sources of information during crises and also enable them to receive information more quickly. However, receiving excessive negative information can provoke negative emotions such as panic and anxiety, thereby reducing public psychological resilience.

#### 2.1.2. Shorten the psychological distance between the public and the crisis

The development of the digital economy can lead to improvements in digital infrastructure. Digital imaging and virtual reality technologies can more realistically reproduce the situation in the affected areas, something that was not possible in the era of traditional media. Therefore, the development of the digital economy enhances the public’s empathic ability toward crises, thereby narrowing the psychological distance between the public and the crisis. According to the relevant ripple effect theory, the closer people are to the center of the crisis, the stronger their negative emotions, which in turn reduces their psychological resilience (16).

Based on this, this paper proposes Hypothesis 1:

*H1*: The spatial direct effect of digital economy development on public psychological resilience is negative.

### 2.2. Indirect effect channels

The digital divide brought about by the development of the digital economy has been shown by some studies to potentially exacerbate regional development imbalances. According to intergroup threat theory in psychology, if groups are divided geographically into in-groups and out-groups, the development of the digital economy in external regions will increase the development gap between regions, further deepening the distinction between in-groups and out-groups. This will cause the in-group to feel a stronger threat from the out-group, thereby generating a positive psychological resistance within the in-group (17). In this way, the development of the digital economy in external regions indirectly promotes the psychological resilience of the public in the internal regions.

Based on this, this paper proposes Hypothesis 2:

*H2*: The spatial indirect effect of digital economy development on public psychological resilience is positive.

## 3. Research method

### 3.1. Global principal component analysis

One of the common methods for determining the weights of an indicator system is the principal component analysis method. The core idea of principal component analysis is to reduce multiple indicators into several uncorrelated composite indicators based on the sample’s covariance matrix or correlation matrix. Each composite indicator is a linear combination of the original indicators, and is expressed as follows:


$$\{\begin{array}{c} Y_{1}=u_{11}X_{1}+u_{21}X_{2}+\cdots u_{p1}X_{p}\\ Y_{2}=u_{12}X_{1}+u_{22}X_{2}+\cdots u_{p2}X_{p}\\ \vdots \\ Y_{p}=u_{1p}X_{1}+u_{2p}X_{2}+\cdots u_{pp}X_{p} \end{array}$$

where $X_{i}$ represents the p original indicators of the data; $Y_{i}$ represents the p composite indicators after principal component analysis; $u_{ij}$ is the coefficient.

Select the top principal components that have a stronger ability to explain the original data based on the variance contribution rate and eigenvalues. Obtain the coefficients of the standardized indicator variables’ linear combination by dividing the factor loading by the square root of the corresponding eigenvalue. Treat the coefficients as weights for the original indicator variables. Principal component analysis balances objectivity and practicality, avoiding partiality like entropy weighting methods and randomness like subjective weighting methods. Traditional principal component analysis is generally for cross-sectional data, while optimized principal component analysis, such as the time series global principal component analysis method, is used for panel data. This method incorporates the characteristics of time series analysis and global principal component analysis to replace the original global variables with a comprehensive variable, thereby overcoming the limitation of traditional principal component analysis, which has no comparability between different time periods (18).

### 3.2. Spatial econometric model

Before building a spatial econometric model, it is necessary to conduct Exploratory Spatial Data Analysis (ESDA) to investigate the spatial relationship between geographical location and variables from a geographical perspective (19), in order to preliminary examine whether both the dependent and independent variables have spatial relevance. The commonly used calculation index is the Global Moran’s Index, with the following calculation formula:


$${\mathrm{Moran}}^{\prime }s\;I=\frac{N}{S_{0}}\frac{\sum_{i=1}^{N}\sum_{j=1}^{N}\omega_{\mathrm{ij}}\left(x_{i}-\bar{x}\right)\left(x_{j}-\bar{x}\right)}{\sum_{i=1}^{N}{\left(x_{i}-\bar{x}\right)}^{2}}$$

where N is the number of observed samples in the spatial unit, $x_{i}$ is the observed value of variable x in spatial unit $i,\bar{x}$ is the mean value of variable x in the spatial unit, $\omega_{\mathrm{ij}}$ is the element of the spatial weight matrix corresponding to spatial units *i* and *j*, $S_{0}$ is the sum of all elements in the spatial weight matrix, with the following calculation formula:


$$S_{0}=\overset{N}{\underset{i=1}{\sum }}\overset{N}{\underset{j=1}{\sum }}\omega_{\mathrm{ij}}$$

Spatial econometric models are classified into three types: spatial autoregressive models, spatial error models, and spatial Durbin models. Before choosing a model, exploratory spatial data analysis must be conducted to examine the spatial correlation between the explanatory variables and the dependent variable. The spatial Durbin model is a model that includes both explanatory and dependent variables with a spatial lag term. When setting up the model, LM and LR tests must be performed to ensure non-degeneracy. For models based on panel data, a Hausman test must be performed to determine if the model adopts a fixed effect or random effect.

## 4. Data acquisition and index system

### 4.1. Instructions on data collection of mental toughness

The commonly used methods for collecting data on psychological resilience are divided into static and dynamic methods. Static methods mainly collect data in the form of questionnaires using relevant psychological resilience scales, while dynamic methods depict and describe the public’s psychological response and spread through simulation and imitation (20–22). Considering the advantages of both static and dynamic methods combined with the characteristics of psychological resilience, the use of Baidu search index to depict psychological resilience data is attempted for the following reasons: as an intrinsic characteristic, public psychological resilience needs to be reflected through a certain medium. Combined with the analysis above, when faced with a crisis event with a high degree of diffusion, the public will obtain information through certain channels. Previous research has shown that when psychological resilience is low, negative psychological emotions have a greater impact and increase the public’s demand for information (23). Baidu search index reflects the number of times that netizens in each province actively search for keywords, directly embodying the public’s demand for information, and thus can serve as a variable that reflects psychological resilience. Baidu search index is high-frequency data. To eliminate the influence of short-term interfering factors on Baidu search index, the overall daily average of Baidu search index is used instead of the fluctuation amplitude of Baidu search index to measure public psychological resilience.

### 4.2. Index system of psychological resilience

**Figure 1:**
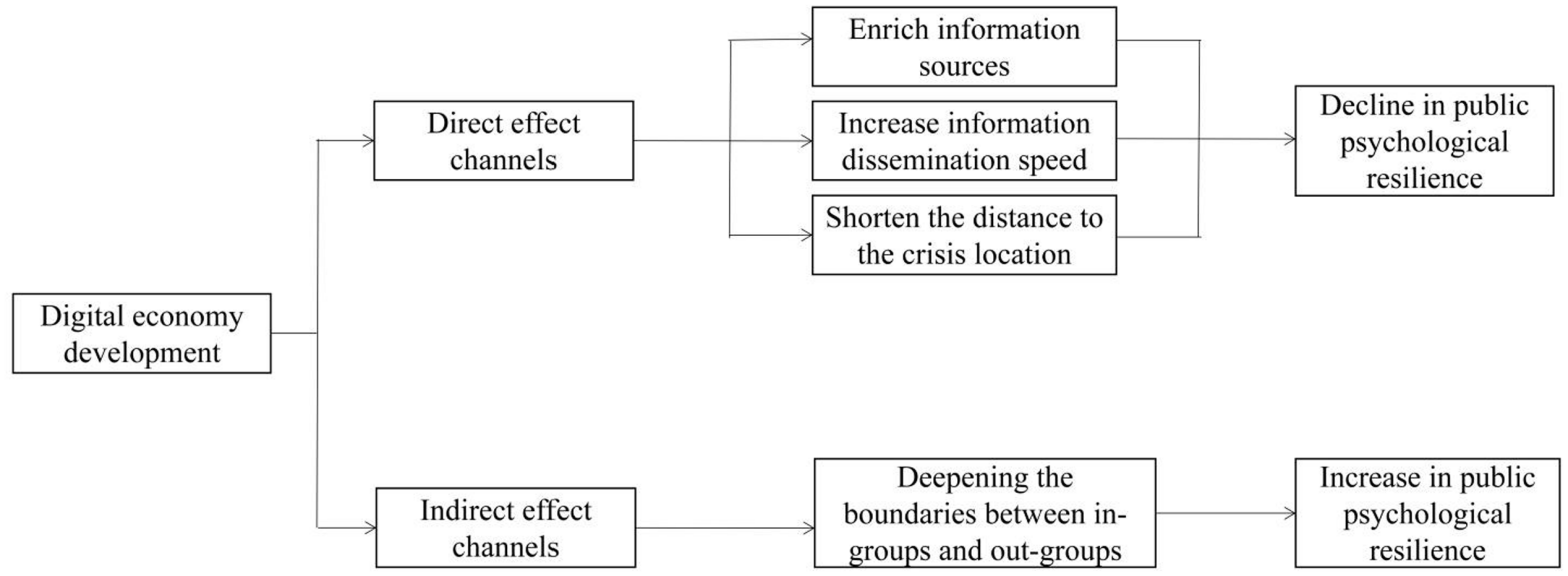
The channel action mechanism chart of the development level of digital economy on public psychological resilience.

Based on the definition of psychological resilience, combined with the commonly used scales of psychological resilience, and referring to the research conclusions of Wang et al. and Wen et al., a psychological resilience indicator system was constructed based on the scientific and comprehensive design of the indicator system and the principle of data availability (see Table 1) (24–26). It includes four commonly seen public psychological emotions of panic, anxiety, sadness, and optimism as primary indicators and 8 secondary indicators that are divided into detail. In order to ensure that the data can reflect the diversity and complexity of public psychology in a comprehensive and accurate manner, the overlapping method of key words is adopted when selecting Baidu Index (Figure 1).

**Table 1:** Index system of psychological resilience.

Level 1 indicators	Level 2 indicators	Baidu search overlay keywords	Attributes	Weights
Panic (P)	Protection panic(P_1_)	Disinfection, medication, masks	Negative direction	0.1213
	Panic situation(P_2_)	Price index, road closure	Negative direction	0.1299
Anxiety (A)	Material anxiety(A_1_)	Panic purchase of salt, panic purchase of rice	Negative direction	0.1135
	Environmental anxiety(A_2_)	Diffusion, infectious diseases	Negative direction	0.1312
Sadness (S)	Internal grief(S_1_)	Sequelae, symptoms	Negative direction	0.1251
	Empathize with grief(S_2_)	Mourning, sacrifice, death toll	Negative direction	0.1303
Optimism (O)	Optimism on society(O_1_)	Rescue, release	Positive direction	0.1247
	Achievement optimism(O_2_)	Donation	Positive direction	0.1239

Before determining the weights of the indicator variables using the sequential global principal component analysis, the negative indicators in the indicator system must be normalized into positive ones, considering the actual meaning represented by the indicator variables, using the following formula:


$$x_{ij}^{\prime }=\frac{\max\left(x_{ij}\right)-x_{ij}}{\max\left(x_{ij}\right)-\min\left(x_{ij}\right)}$$

where $x_{ij}^{\prime }$ represents the index value after forward processing; $x_{ij}$ represents the original index data value; $\max\left(x_{ij}\right)$ and $\min\left(x_{ij}\right)$ are, respectively, the maximum and minimum values in the index data.

There are two approaches to extracting principal components in the main component analysis, one is to solve the eigenvalues from the covariance matrix, and the other is to solve the eigenvalues from the correlation matrix. This paper starts with the correlation matrix to extract the principal components, which eliminates the process of index standardization and obtains the contribution rate of each principal component to the variance and the coefficients of linear combinations of each principal component, which can fully reflect the ratio of the information contained in each principal component to the original data and the total information, making the use of principal component analysis more objective and reasonable in assigning weights. After the main component analysis iteration, the variance explanation rate shows that a total of 1 eigenvalue greater than 1 linear combination was extracted as the main component, but considering the load coefficients and the interpretation of the actual meaning of the main component, finally two linear combinations were extracted as the main components, which accumulated 92.219% of the original data information from 2011 to 2020 (see Table 2), in line with the requirements of the temporal global principal component analysis. Firstly, the factor loading is divided by the square root of the corresponding eigenvalue to obtain the coefficient of the standardized index variable linear combination. Finally, the comprehensive evaluation model of public psychological resilience can be obtained by multiplying the variance explanation rate with the scoring coefficients of each principal component and adding them up, and dividing by the cumulative variance explanation rate.


$$\begin{array}{l} \mathrm{Composition}=0.3305P_{1}+0.3542P_{2}+0.3096A_{1}+0.3579A_{2}+\\ 0.3413S_{1}+0.3554S_{2}+0.3401O_{1}+0.3380O_{2} \end{array}$$

**Table 2:** Public mental resilience principal component analysis variance explanation table.

Serial number	Characteristic root	Variance explanation rate%	Cumulative variance explanation rate%
1	6.593	82.411	82.411
2	0.785	9.807	92.219

The weights of the secondary indicators correspond to the normalized values of the linear combination coefficients in the comprehensive evaluation model. To eliminate the influence of the original data’s unit of measurement on the indicators, the original data is standardized using *Z*-score normalization. The public psychological resilience measurement value can be obtained by multiplying the weights determined by the secondary indicators after eliminating the unit of measurement with the corresponding data.


$$PR_{i}=\overset{8}{\underset{i=1}{\sum }}\omega_{i}*X_{i}^{*}$$

where $\omega_{i}$ is the weight of each secondary index; $X_{i}^{*}$ is the secondary index value transformed by -score standardization.

The green development level score obtained is processed using the second type efficiency coefficient method, which restricts the data range to [40,100] and has good interval stability (27).


$$PR_{i}^{*}=40+60\times \frac{PR_{i}-\min\left\{PR_{i}\right\}}{\max\left\{PR_{i}\right\}-\min\left\{PR_{i}\right\}}$$

### 4.3. Digital economy development level index system

Under the guidance of the digital economy connotation idea published in the “White Paper on the Development of China’s Digital Economy (2020)” by China Institute of Communications, the digital economy development level index system is constructed in accordance with the principles of systematization, completeness, and scientifically of the index system, based on the practice of Li et al. (28, 29). In order to better reflect the impact of the change in communication media brought about by the development of the digital economy on public psychological resilience during the diffusion of crisis events, not only the explicit impact of digital infrastructure, but also the implicit impact of factors such as the digital economy industry and digital environment, needs to be considered. Therefore, the index system includes three aspects: digital carrier, digital industry, and digital environment, and the indicators of the three aspects are refined into 9 secondary indicators (see Table 3). All indicators in the index system are positive indicators, meaning that the higher the value of the indicator, the higher the level of digital economic development.

**Table 3:** Digital economy development level index system.

Level 1 indicators	Level 2 indicators		Attributes	Weights
Digital carrier (C)	Fiber optic cable length(C_1_)	kilometers	Positive direction	0.0246
	Number of mobile phone base stations(C_2_)	10 thousand	Positive direction	0.0511
	Number of Internet broadband access ports(C_3_)	10 thousand	POSITIVE direction	0.0477
Digital industry (I)	Share of income from information service industry and software industry(I_1_)	%	Positive direction	0.1602
	Proportion of computer services and software employees(I_2_)	%	Positive direction	0.1606
	*per capita* telecom traffic(I_3_)	100 million yuan per ten thousand people	Positive direction	0.1122
Digital environment (E)	Internet penetration rate(E_1_)	%	Positive direction	0.1581
	Mobile phone penetration rate(E_2_)	%	Positive direction	0.1675
	Digital inclusive financial index(E_3_)	/	Positive direction	0.1180

The proportion of income from computer services and software industry is equal to the ratio of the sum of computer services and software industry income to the regional GDP. The digital inclusive finance index uses Peking University’s digital inclusive finance index.

The determination method of the weight of the digital economy development level is the same as the determination method of the weight of public psychological resilience. Through the temporal global principal component analysis method, two linear combinations with eigenvalues greater than 1 are extracted as the main components. During the period of 2011–2020, a total of 81.104% of the original data information was accumulated (see Table 4), which meets the standards of principal component analysis. The standardized index variables linear combination coefficient is obtained by dividing the factor loading by the square root of the corresponding eigenvalue. The mathematical expression of the score linear combination of the two main components is:


$$\begin{array}{l} \mathrm{Composition}1=0.292C_{1}+0.339C_{2}+0.325C_{3}+0.283I_{1}+0.273I_{2}+\\ 0.334I_{3}+0.380E_{1}+0.368E_{2}+0.385E_{3} \end{array}$$

$$\begin{array}{l} \;\mathrm{Composition}2=-0.461C_{1}-0.391C_{2}-0.384C_{3}+0.414I_{1}+0.439I_{2}+\\ 0.005I_{3}+0.196E_{1}+0.283E_{2}-0.067E_{3} \end{array}$$

**Table 4:** Digital economy development level principal component analysis variance explanation table.

Serial number	Characteristic root	Variance explanation rate%	Cumulative variance explanation rate%
1	4.951	55.016	55.016
2	2.348	26.088	81.104

The comprehensive evaluation model of the digital economic development level can be obtained by multiplying the variance explanation rate with the coefficients of each principal component score and adding them up, then dividing by the cumulative variance explanation rate.


$$\begin{array}{l} \mathrm{Composition}=0.0499C_{1}+0.1039C_{2}+0.0970C_{3}+0.3255I_{1}+0.3263I_{2}+\\ 0.2279I_{3}+0.3212E_{1}+0.3404E_{2}+0.2398E_{3} \end{array}$$

The final measurement value is obtained by still using the efficiency coefficient method mentioned earlier to process the result.

## 5. Empirical analyses

### 5.1. Analysis on the measurement of public psychological resilience and the development level of digital economy

First, based on the characteristics of the measurement value and the actual situation of public psychological resilience and digital economic development, the measurement value is divided into different levels. Due to the strong central tendency of the public psychological resilience measurement value dataset, it is not convenient for subjective division of levels. Therefore, following Le Gallo J’s suggestion, the measurement value is divided into four states based on the quartile principle with 25%, 50%, 75% as the boundary (30), that is, the measurement value is divided into non-overlapping complete intervals [40, 86.20], [86.20, 90.76], [90.76, 94.07], [94.07, 100] by dividing the boundary into intervals. As the indices are all processed in a positive direction, the higher the measurement value, the stronger the public psychological resilience. Therefore, the four levels are named in order as Level 1 for poor, Level 2 for general, Level 3 for good, and Level 4 for strong. Similarly, since the discrete trend of digital economic development level measurement value is relatively obvious, the method of subjective division of levels is adopted, that is, the measurement value above 70 points is considered a very developed province, between 60 and 70 is a relatively developed province, between 50 and 60 is a generally developed province, and below 50 points is considered a less developed province. Due to the limited space, only the measurement values of public psychological resilience and digital economic development in 2013, 2016, 2020, and the average level from 2011 to 2020 are listed and displayed (see Table 5).

**Table 5:** Measure of public psychological resilience (PPR) and digital economy development level (DEDL) in some years.

Province	2013		2016		2020		Average level		Level	
	PPR	DEDL	PPR	DEDL	PPR	DEDL	PPR	DEDL	PPR	DEDL
Beijing (BJ)	85.95	69.17	78.99	74.28	63.28	93.25	78.19	76.07	1	2
Tianjing (TJ)	93.45	51.93	92.11	55.80	86.27	70.12	91.47	57.12	3	1
Hebei (HF)	92.18	49.04	90.25	53.39	97.28	63.79	89.83	53.45	2	1
Shanxi (SX)	94.87	49.14	93.06	52.45	84.62	62.89	92.07	52.98	3	1
Inner Mongolia (IM)	97.49	50.56	95.47	52.51	89.16	63.38	94.85	53.73	4	1
Liaoning (LN)	93.32	53.97	90.84	56.65	80.61	66.46	88.99	57.29	2	1
Jilin (JL)	96.28	49.11	94.03	53.22	87.66	63.05	93.24	53.47	3	1
Heilongjiang (HL)	95.84	47.69	91.76	52.19	85.59	62.93	92.27	52.29	3	1
Shanghai (SH)	91.71	60.63	82.38	63.11	66.90	80.24	82.28	65.30	1	1
Jiangsu (JS)	86.82	54.28	82.06	58.47	58.02	70.63	79.20	59.21	1	1
Zhejiang (ZJ)	86.57	56.59	81.52	61.07	59.63	74.32	79.03	62.15	1	1
Anhui (AH)	93.71	46.13	91.82	50.41	75.67	61.44	88.96	50.77	2	1
Fujian (FJ)	91.41	54.22	89.28	56.91	76.38	66.89	87.61	57.84	2	1
Jiangxi (JX)	95.17	45.44	93.01	49.42	81.40	59.66	91.02	49.74	3	1
Shandong (SD)	89.38	50.29	85.16	55.35	63.75	65.45	82.18	55.12	1	1
Henan (HN)	91.37	46.75	88.72	51.53	71.37	61.88	86.31	51.34	2	1
Hubei (HE)	90.37	48.41	87.71	52.42	72.16	63.25	85.70	52.90	1	1
Hunan (HA)	91.99	46.55	90.47	50.25	77.18	61.28	88.13	50.87	2	1
Guangdong (GD)	81.64	58.62	74.07	62.80	40.01	75.96	72.18	63.79	1	1
Guangxi (GX)	93.50	45.91	93.59	49.98	83.07	61.60	91.22	50.74	3	1
Hainan (HI)	98.76	49.35	97.70	52.23	92.10	62.44	97.07	53.30	4	1
Chongqing (CQ)	94.09	48.65	90.96	52.56	79.14	63.41	89.89	53.17	2	1
Sichuan (SC)	90.76	48.94	84.06	54.10	65.77	65.70	83.89	54.09	1	1
Guizhou (GZ)	95.61	45.73	93.84	49.77	86.89	61.26	93.26	50.49	3	1
Yunnan (YN)	95.17	46.40	92.31	49.93	85.24	61.52	92.18	50.59	3	1
Shaanxi (SN)	93.24	49.92	91.10	53.75	81.17	64.91	90.13	54.52	2	1
Gansu (GS)	97.72	46.11	94.78	49.35	89.60	59.93	94.81	50.11	4	1
Qinghai (QH)	99.78	48.88	98.97	50.90	95.57	62.46	98.60	52.52	4	1
Ningxia (NX)	99.62	48.43	98.45	51.54	94.76	61.23	98.17	52.29	4	1
Xinjiang (XJ)	98.04	49.18	96.42	51.11	91.11	62.74	95.58	52.31	4	1

From the temporal characteristics of public psychological resilience and digital economic development, it can be seen that the measurement value of public psychological resilience shows a declining trend in the sample period. On average, there is at least one level of decline. Taking the Yangtze River Delta region as an example, Shanghai’s value dropped from 91.71 in 2013 to 66.90 in 2020, from a good level to a poor level; similarly, Zhejiang Province’s value dropped from 86.57 in 2013 to 59.63, from a general level to a poor level. This indicates that the public’s ability to self-regulate and adapt to spreading crisis events has significantly weakened over time. This change may be related to the public’s increasing negative psychological emotions and declining ability to cope with crisis events when facing increasingly intense life pressures. The level of digital economic development, on the other hand, has significantly increased and the overall growth rate is relatively fast, demonstrating the strong growth potential of the digital economy. This achievement is due to the support of national policies for the information and digital industry, the improvement of digital infrastructure and the widespread application of digital technology.

**Figure 2:**
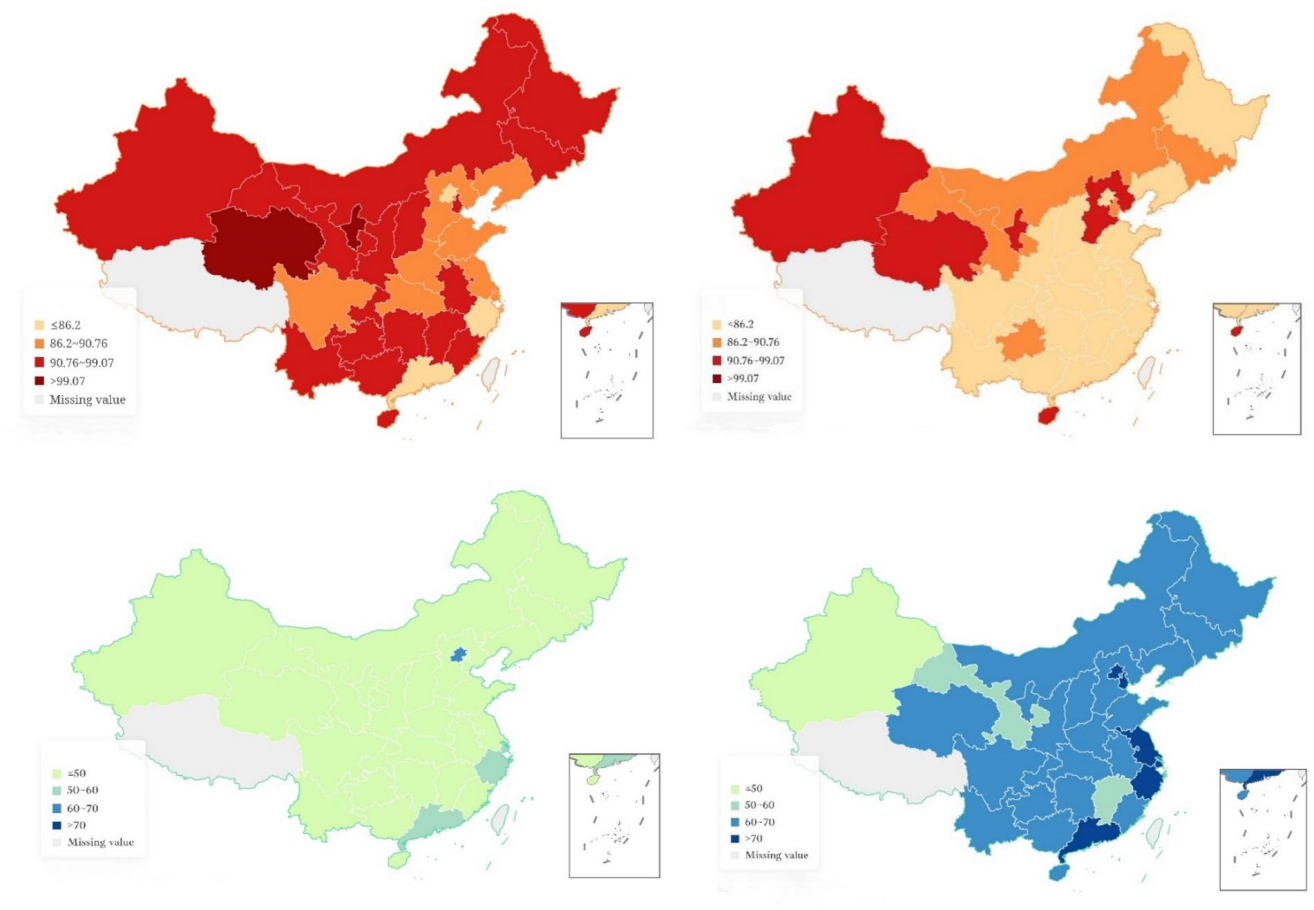
Spatial distribution of public psychological resilience and digital economy development level measurement values in 2011 and 2020. The left and right sides represent the years 2011 and 2020, respectively, while the top and bottom represent PR and Dige, respectively.

It can be seen from the spatial characteristics of public psychological resilience and digital economic development that regions with low values of public psychological resilience are concentrated in Beijing, the Yangtze River Delta, and the southeast coastal areas, while regions with high values of public psychological resilience are mainly concentrated in the northwest. Correspondingly, regions with high values of digital economic development level are concentrated in Beijing, the Yangtze River Delta, and the southeast coastal areas, while regions with low values of digital economic development level are concentrated in central regions. This shows that the regions with low public psychological resilience are also the regions with high values of digital economic development, indicating that the development of the digital economy may have a negative impact on public psychological resilience through new digital media, and therefore the two may have a spatial association. In conclusion, both public psychological resilience and digital economic development level have certain spatial heterogeneity. Specifically, public psychological resilience has a spatial distribution characterized by decreasing from high in the west, medium in the central, to low in the east, while digital economic development level has a “U” shaped spatial structure with high values in the east and west, and low in the middle (see Figure 2).

### 5.2. Exploratory spatial data analysis

Before conducting exploratory spatial data analysis, it is necessary to first determine the spatial weight matrix to be used. Considering the actual background of the study based on diffusive crisis and combining the results of Su Fang et al.’s research and the theoretical analysis in the previous sections, it is noted that the public’s psychology is influenced by the distance from the event center when faced with crisis events (31). Therefore, the most suitable spatial weight matrix is the spatial geographical distance matrix based on latitude and longitude as the distance measure standard. The specific formulation is as follows:


$$W_{ij}=\{\begin{array}{c} \frac{1}{D_{ij}}\;i\neq j\\ \;0\;i=j \end{array}$$

where $D_{ij}$ represents the geographical distance between i and j calculated by latitude and longitude.

In order to further reveal the spatial correlation laws, based on this weight matrix, the global Moran index of the public psychological resilience and digital economic development level measurement values is calculated (see Table 6). It can be seen that the global Moran index of public psychological resilience and digital economic development level measurement values from 2011 to 2020 are significantly positive at a significance level of 5%, indicating that public psychological resilience and digital economic development level both have significant positive spatial correlation. That is, the rise of corresponding indicators in one region will cause the rise of corresponding indicators in geographically adjacent regions, reflecting the strong positive radiation effect of the geographical center region on public psychological resilience and digital economic development level. From a temporal perspective, the change of the global Moran index of public psychological resilience and digital economic development level is relatively small, indicating that the spatial correlation between public psychological resilience and digital economic development level is not accidental, and has certain stability.

**Table 6:** The global morale index of the public’s psychological resilience and the level of digital economic development for the years 2011–2020.

Year	PR		Dige	
	Moran’s I	*p*	Moran’s I	*p*
2011	0.058^***^	0.005	0.048^***^	0.006
2012	0.075^***^	0.001	0.044^***^	0.008
2013	0.045^∗∗∗^	0.013	0.033^**^	0.021
2014	0.067^***^	0.002	0.028^**^	0.027
2015	0.046^**^	0.012	0.028^**^	0.027
2016	0.026^*^	0.045	0.038^**^	0.013
2017	0.051^***^	0.009	0.039^**^	0.013
2018	0.049^***^	0.010	0.028^**^	0.028
2019	0.046^**^	0.013	0.034^**^	0.017
2020	0.041^**^	0.017	0.044^***^	0.006

The symbols “ ∗∗∗ “, “ ∗∗ “, and “ ∗ “respectively represent significant levels at 1%, 5%, and 10%, respectively. The same applies throughout.

**Figure 3:**
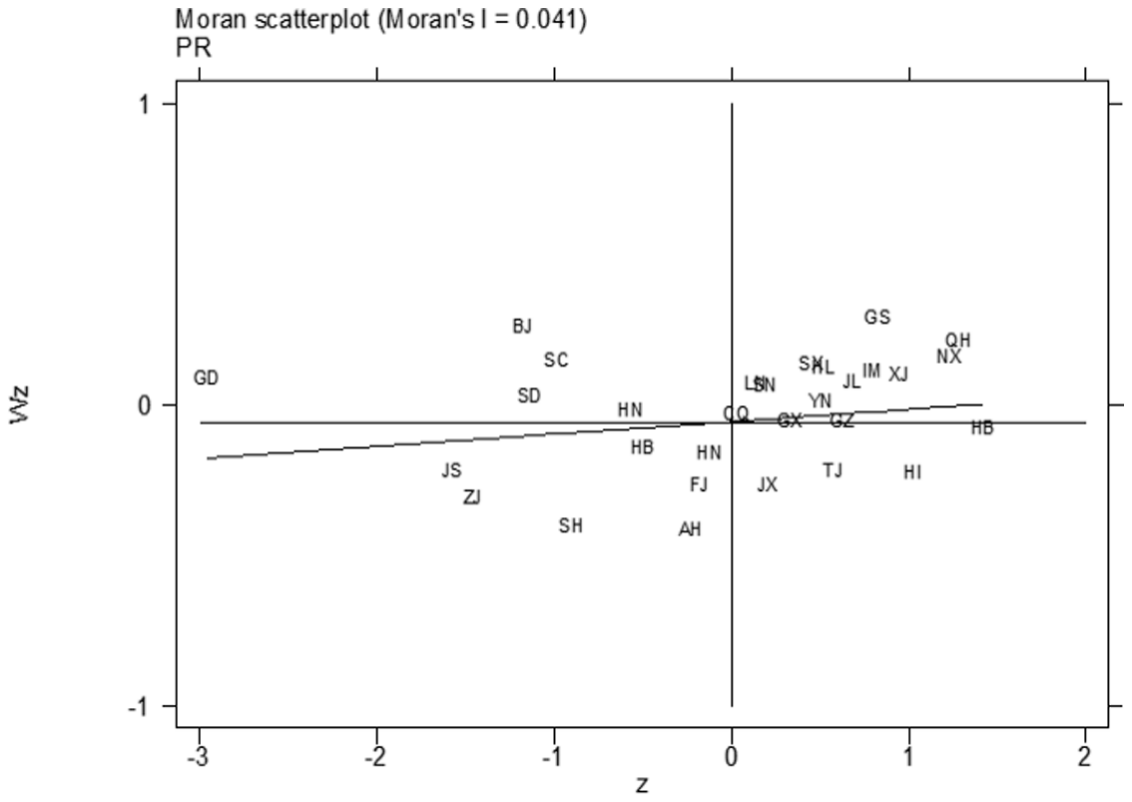
2020 Morans scatter plot of public psychological resilience and digital economic development.

The Global Morale Index can only judge the spatial correlation between public psychological resilience and the level of digital economic development on a global scale. However, the spatial dependence between different provinces still needs to be further verified. Based on this, a Morale Scatterplot of the public psychological resilience and the level of digital economic development in 2020 is drawn for the analysis of local spatial correlation (refer to Figure 3). It can be seen that most provinces are in the first and third quadrants, exhibiting spatial distribution characteristics of high value clustering and low value clustering. In terms of public psychological resilience, the provinces in the third quadrant are mainly located in the Yangtze River Delta region and the eastern coastal provinces, indicating that for relatively developed economic regions, public psychological resilience shows low clustering effect and is far from the origin, forming a relatively obvious low-value clustering area. However, for the level of digital economic development, the Yangtze River Delta region and the eastern coastal provinces are mainly located in the first quadrant, indicating that for relatively developed economic regions, unlike public psychological resilience, the level of digital economic development shows a significant high-high clustering effect. The provinces in the third quadrant, which are closer to the origin and have a more clustered tendency than public psychological resilience in the figure, indicate that compared with public psychological resilience, the level of digital economic development has not yet formed a relatively obvious low-value clustering area. The analysis of the local spatial correlation between the two further reflects the spatial heterogeneity of the indicators in the eastern, central, and western provinces.

### 5.3. Model setting and model testing

Based on the above analysis, this paper’s dependent variable is the public psychological resilience ( PR ) measured in the previous section; the core explanatory variable is the level of digital economy development ( Dige ) measured previously; considering the relationship between public psychological resilience and the level of digital economy development and other exogenous variables that may affect public psychological resilience, referring to existing research on the factors affecting public psychological resilience, the conclusion is that when choosing control variables, one should include indicators that reflect economic changes, and on the other hand, indicators that reflect individual differences should be included (32, 33). In summary, the following indicators were selected as control variables: *per capita* disposable income ( NI ), *per capita* regional gross domestic product ( PGDP ), regional unemployment rate ( UR ), and regional *per capita* years of education ( EDU ; see Table 7).

**Table 7:** Model variable setting and description.

Variable	Variable symbol	Meaning	Description
Explained variable	$PR_{it}$	Public psychological resilience	Measure values obtained by time-series global principal component analysis
Core explanatory variable	$Dige_{it}$	Digital economy development level	Measure values obtained by time-series global principal component analysis
Control variable	$NI_{it}$	*per capita* disposable income of regional residents	
	$PGDP_{it}$	Gross regional product *per capita*	Regional GDP / Regional population
	$UR_{it}$	Regional unemployment rate	
	$EDU_{it}$	Years of schooling *per capita* in the region	(Number of illiterate people*0 + number of people with elementary school education*6 + number of people with junior high school education*9 + number of people with high school and junior college education*12 + number of people with college and bachelor’s degree or higher education*16)/total population over 6 years old

Based on the analysis of the research methods and the selection of indicator variables, a spatial econometric model is constructed with the spatial Durbin model as the basis. The model includes the spatial lag terms of both the explanatory variables and the explained variables. All variables are logged to eliminate the impact of scale, making the data comparable. At the same time, the parameter meaning in the explanation model is elasticity, i.e., the size of the corresponding change in the explained variable when the explanatory variable changes by one percentage point.

Before setting the model, LM tests and robust LM tests are required to assist in the setting of the model form (see Table 8). The LM test, also known as the Lagrange Multiplier test, is a pre-test method. From the test results, the spatial error term and the spatial lag term are significant at the 5% significance level, indicating that either a spatial autoregressive model or a spatial error model can be selected. Therefore, a general form spatial Durbin model that includes both is the most appropriate. The specific form of the model setup is as follows:


$$\begin{array}{l} \ln{\mathrm{PR}}_{\mathrm{it}}=\alpha +\rho {\mathrm{WlnPR}}_{\mathrm{it}}+\beta ln{\mathrm{Dige}}_{\mathrm{it}}+\delta lnX_{\mathrm{it}}+\\ {\mathrm{\theta WlnDige}}_{\mathrm{it}}+\sigma {\mathrm{WlnX}}_{\mathrm{it}}+u_{i}+\gamma_{i}+\varepsilon_{\mathrm{it}} \end{array}$$

Where, $X_{\mathrm{it}}$ is a series of control variables; $u_{i}$ is the regional effect; $\gamma_{i}$ is the time effect; $\varepsilon_{\mathrm{it}}$ is the random disturbance term.

**Table 8:** LM test and robust LM test.

Inspection name	Statistical value	*p*
LM-error	109.365	0.000
Robust LM-error	135.273	0.000
LM-lag	0.472	0.492
Robust LM-lag	26.381	0.000

After determining the form of the model specification, it is necessary to examine whether the model should adopt regional fixed effects, time fixed effects, or both. By comparing the likelihood values and goodness of fit of different models, the time fixed effects model was finally selected. The Hausman test is commonly used to determine whether the model should adopt fixed or random effects. The results show that the Hausman test statistic is 25.37, and the *p*-value is 0.0001, which is less than 0.05, indicating that the fixed effects model is preferred by rejecting the null hypothesis. After preliminary determination of the model form, post-hoc Wald test and LR test are still necessary to ensure that the spatial Durbin model will not degenerate into other models, thus ensuring the stability of the spatial Durbin model (see Table 9). The test *p*-values are both 0.000, indicating extremely significant results and rejecting the null hypothesis, which means that the spatial Durbin model will not degenerate into the spatial autocorrelation model and spatial error model. In conclusion, the spatial Durbin model with time fixed effects is the optimal choice.

**Table 9:** Wald test and LR test.

Inspection name	Statistical value	*p*
Wald Test for SAR	85.19	0.000
Wald Test for SEM	81.88	0.000
LR Test for SAR	59.08	0.000
LR Test for SEM	59.28	0.000

### 5.4. Analysis of spatial econometric model results

The spatial Durbin model’s regression results (refer to Table 10) show that at a 1% significance level, the coefficient of the core explanatory variable, digital economic development level, is significantly negative, indicating that a higher digital economic development level has a negative effect on public psychological resilience in the area, meaning that the higher the digital economic development level in the province, the lower the public psychological resilience. The spatial lag term of the digital economic development level is significantly positive at the 5% significance level, indicating that the digital economic development level of surrounding areas has a positive spillover effect on local public psychological resilience. The use of the spatial Durbin model to judge spatial effects may have some bias, so the LeSage J’s approach was used to decompose the spatial effects into direct and indirect effects using partial differential methods to eliminate systematic bias and obtain the true effect of spatial effects (34). The direct effect of digital economic development level is significant and negative, while the indirect effect is significant and positive, indicating that local digital economic growth will suppress local public psychological resilience, but differently, the development of digital economy in surrounding areas will promote local public psychological resilience through positive spillover effects. The reason for this phenomenon may be that, according to the “psychological eye of the storm effect” and information security perspectives, when facing diffuse crisis events, each region can be considered as a crisis center, and the closer the geographic distance from the crisis center, the stronger the negative emotions generated by the public, thus reducing public psychological resilience. On the other hand, the amount of information obtained and its impact on public psychological resilience is often a double-edged sword. The development of the digital economy has expanded the sources of information for the public and increased opportunities for information acquisition, but it has also brought about a large amount of negative and false information mixed in with countless fragmented information. According to the recipient-centered theory in communication, negative information is a common warning system and is more easily noticed by the public than positive or neutral information. As a result, the digital economy, as an amplifier of information, has brought more negative information to the public, causing anxiety, panic, and other negative emotions. The effect of the combination of geographical distance and information dissemination ultimately reduces the psychological resilience of the local public (35–37). The positive spatial spillover effect of the digital economy in the surrounding area on the psychological resilience of the local public is related to the social identity and intergroup threat theory in psychology. In psychology, according to different criteria, people can be divided into in-group and out-group, and geographical boundaries are one of the common criteria for dividing the in-group and out-group. The social identity theory suggests that changes in economic, social, and political factors can lead to the appearance of intergroup threat, which leads to changes in intergroup relations and increased in-group bias and out-group prejudice. As the economic development level of the surrounding areas increases, it can lead to intergroup threat to the local area, causing changes in the intergroup relationship between the local area and the surrounding areas. This leads to an increase in social identification and support for the local area, which has been shown to have a positive correlation with psychological resilience. Therefore, facing a spreading crisis, the public in the local area will have increased psychological resilience (38–40).

**Table 10:** Coefficient estimation and effect decomposition of spatial Dubin model.

Variable	Coefficient	Direct effect	Indirect effect	Total effect
$Dige_{it}$	−0.637^***^	−0.648^***^ (0.131)	4.244^***^ (1.067)	3.596^***^ (1.117)
$NI_{it}$	0.053	0.051 (0.051)	−0.639^*^ (0.341)	−0.588^*^ (0.347)
$PGDP_{it}$	−0.016	−0.012 (0.029)	−0.515^*^ (0.279)	−0.527^*^ (0.288)
$UR_{it}$	−0.378^*^	−0.037^*^ (0.022)	0.388^**^ (0.163)	0.351^**^ (0.169)
$EDU_{it}$	0.092	0.083 (0.081)	0.239 (0.406)	0.322 (0.397)
$W*Dige_{it}$	4.302^***^			
$W*NI_{it}$	−0.677^*^			
$W*PGDP_{it}$	−0.504^**^			
$W*UR_{it}$	0.385^***^			
$W*EDU_{it}$	0.228			
$R^{2}$	0.228			
log−likehood	426.4082			

Standard error in brackets.

### 5.5. Regional spatial effect analysis

Due to the significant regional differences and concentration of economic development levels within regions in China, the development patterns of the eastern, central, and western regions are not entirely identical. To further analyze the conclusions obtained earlier, it is necessary to examine whether the selection of different sample regions would lead to different results. Thus, this study divides the 30 provinces into eastern, central, and western regions, and employs the aforementioned spatial Durbin model for regional empirical analysis (see Table 11).

**Table 11:** Regional spatial effect analysis.

Dependent variable: public psychological resilience $\;PR_{it}$			
	Eastern region	Central region	Western region
Direct effect			
$Dige_{it}$	−0.272 (0.250)	0.184 (0.163)	−1.155^***^ (0.199)
$NI_{it}$	−0.123 (0.109)	0.378^***^ (0.075)	−0.300^***^ (0.079)
$PGDP_{it}$	−0.036 (0.052)	−0.227^***^ (0.037)	0.067 (0.056)
$UR_{it}$	−0.042 (0.379)	−0.053^***^ (0.037)	−0.012 (0.022)
$EDU_{it}$	0.687^***^ (0.185)	0.183 (0.161)	0.415^***^ (0.095)
Indirect effect			
$Dige_{it}$	3.286^***^ (0.459)	0.686 (0.651)	1.165^*^ (0.725)
$NI_{it}$	−0.777^**^ (0.381)	−1.002^***^ (0.391)	−0.772^**^ (0.335)
$PGDP_{it}$	0.106 (0.293)	0.197 (0.189)	−0.224 (0.191)
$UR_{it}$	0.564^***^ (0.087)	0.049 (0.144)	−0.126^*^ (0.071)
$EDU_{it}$	−0.116 (0.264)	0.451 (0.619)	1.732^***^ (0.652)
Total effect			
$Dige_{it}$	3.014^***^ (0.523)	0.870 (0.706)	0.011 (0.835)
$NI_{it}$	−0.900^**^ (0.371)	−0.624 (0.434)	−1.072^***^ (0.361)
$PGDP_{it}$	0.071 (0.294)	−0.031 (0.204)	−0.157 (0.208)
$UR_{it}$	0.605^***^ (0.101)	−0.005 (0.174)	−0.137^*^ (0.082)
$EDU_{it}$	0.570^***^ (0.163)	0.633 (0.744)	2.147 (0.706)

The symbols “ ∗∗∗ “, “ ∗∗ “, and “ ∗ “respectively represent significant levels at 1%, 5%, and 10%, respectively. The same applies throughout.

First is to observe the direct effect of the core explanatory variable Dige. For the regional analysis, only the western region’s direct effect is significantly negative at the 1% confidence level, while the eastern and central regions’ direct effects are not significant. This indicates that the improvement of digital economy development level in a province in the western region has a negative impact on the public psychological resilience in that region, while there is no significant impact in the eastern and central regions. Next step is to examine the indirect effect of the core explanatory variable Dige. For the regional analysis, both the eastern and western regions have positive indirect effects at their respective significance levels, while the central region’s direct effect is not significant. This suggests that the digital economy development of neighboring provinces in the eastern and western regions promotes the improvement of public psychological resilience in the respective regions, while the central region does not have a significant impact. Overall, the digital economy development in the eastern region primarily relies on spatial indirect effects to impact public psychological resilience. The digital economy development in the central region does not have a significant impact on public psychological resilience at the spatial effect level. The spatial effect mechanism of digital economy development in the western region is similar to that observed in the full sample analysis. The possible reason for these regional differences is that most provinces in the eastern region have a higher level of digital economy development and possess a more comprehensive digital infrastructure. This means that when facing sudden, contagious public crises, local residents can quickly receive crisis signals and make corresponding psychological adjustments. Therefore, even if the digital economy development level improves, it does not cause local residents to receive information exceeding their psychological expectations during a crisis, and consequently does not directly lead to a decrease in public psychological resilience. On the other hand, in the eastern cities that have long enjoyed the benefits of the digital economy, the digital economy mostly has positive impacts, and the public has already acquired the ability to discern in the face of crisis information, effectively avoiding negative psychological effects brought about by digital information. In the central region, where the digital economy development level is relatively low and the corresponding digital infrastructure lags, information transmission nodes are more dispersed, and various factors contribute to the difficulty of the digital economy to significantly impact public psychological resilience through spatial breakthroughs. As for the western region, most provinces also have relatively lagging digital economy development levels, but there are some provinces with better digital economy development. Therefore, in the context of such imbalanced regional development, it is more likely for a group threat effect to emerge between less developed and better developed areas, thereby producing a mechanism similar to that observed in the full sample analysis. This observation also serves as supplementary evidence that the spatial effect mechanism of digital economy development on public psychological resilience in China is distinct due to the imbalance of regional development.

### 5.6. Robustness test

To enhance the reliability of the conclusions and the credibility of the model, this study employs two methods for robustness testing: replacing the spatial weight matrix and shortening the sample period (see Table 12), which using the spatial adjacency matrix as the spatial weight matrix to construct the spatial Durbin model and reduce the sample data time span to 2014–2020. The comparison results show that the direction and significance level of the core explanatory variables have not changed, nor have their values changed significantly. The overall model fitting effects are satisfactory, indicating that the model has good robustness.

**Table 12:** Robustness test results.

Variables	Original model	Spatial adjacency matrix	2014–2020 data
${\mathrm{Dige}}_{\mathrm{it}}$	−0.637***	−0.618***	−0.796***
$W*{\mathrm{Dige}}_{\mathrm{it}}$	4.302***	1.788***	5.015***
Control variables	Controlled	Controlled	Controlled
Log-likehood	426.4082	441.2223	280.9632

The symbols “ ∗∗∗ “, “ ∗∗ “, and “ ∗ “respectively represent significant levels at 1%, 5%, and 10%, respectively. The same applies throughout.

## 6. Research conclusions and policy recommendations

### 6.1. Research conclusions

The paper uses search index data from Baidu from 2011 to 2020 and panel data from 30 provinces in China to measure public psychological resilience and digital economic development level using a time-series global principal component analysis method. The calculated values are used to establish a spatial Dubin model to empirically analyze the impact of digital economic development level on public psychological resilience in the context of a diffusive crisis. The following conclusions are drawn: First, in terms of the measurement of public psychological resilience and digital economic development level, public psychological resilience shows a spatial distribution feature with high levels in the west, followed by the central regions, and the lowest levels in the east, while digital economic development level shows a “U”-shaped spatial structure feature with high levels in both the east and the west and low levels in the central region. Second, from the perspective of spatial correlation, both public psychological resilience and digital economic development level have significant spatial correlations and there is a certain degree of reciprocity between public psychological resilience and digital economic development level in their spatial distributions, such as the coastal areas in the east being a low-value gathering area for public psychological resilience but a high-value gathering area for digital economic development level. Third, in terms of spatial effects, local digital economic development level has a negative impact on local public psychological resilience, while the digital economic development level of surrounding regions has a positive spatial spillover effect on local public psychological resilience due to social identity and intergroup relationships theories.

### 6.2. Policy recommendations

Based on the relationship between public psychological resilience and the level of digital economic development, some relevant policy recommendations can be made: On the one hand, the government should take advantage of the benefits brought by the digital economy and widely use digital media to release authoritative information during the spread of crisis events, thereby enhancing the public’s ability to judge the situation and meet their need for information, ultimately improving the public’s psychological resilience when dealing with crises. On the other hand, due to the positive spillover effect of the digital economy’s development on public psychological resilience, it is necessary to pay more attention to balanced regional development when developing the digital economy, so that the public in each region can equally enjoy the convenience brought by the digital economy, thereby expanding the scope of the defined internal group and increasing the core cohesion of the regional public, giving full play to the radiation effect of the central region’s digital economy, creating a growth pole for the digital economy, and further improving the psychological resilience of the region under the background of digital economic development.

### 6.3. Research gaps and future perspectives

The current study establishes indicators for the level of digital economic development and public psychological resilience based on China’s current digital economic development plan and Baidu search index. However, limitations exist in the research process, including the single data source for constructing the psychological resilience indicator system, which may cause differences in understanding. Future researches can combine data from multiple databases to create a more comprehensive indicator system. Additionally, all data used in this study are cross-sectional data, which can only analyze the correlation between variables rather than causation. Future longitudinal studies can explore the causal relationship between digital economy and psychological resilience. Digital economy is a dynamic and multidimensional concept, and the continuous update of digital technology will greatly enrich its connotation. Similarly, people’s preferred Baidu search terms during different diffusion crises are not completely identical, so the measurement of psychological resilience also needs dynamic improvement. Therefore, this study can measure and analyze the development of the digital economy and public psychological resilience from different perspectives, and conduct empirical analysis of their spatial effects, thus analyzing different results mechanisms at different stages and forming more timely policy recommendations.

## Data availability statement

The original contributions presented in the study are included in the article/supplementary material, further inquiries can be directed to the corresponding author.

## Author contributions

ZW conceived this study. JT collected the data and wrote the draft. JT, ZW, and JL revised the draft. All authors contributed to the article and approved the submitted version.

## Conflict of interest

The authors declare that the research was conducted in the absence of any commercial or financial relationships that could be construed as a potential conflict of interest.

## Publisher’s note

All claims expressed in this article are solely those of the authors and do not necessarily represent those of their affiliated organizations, or those of the publisher, the editors and the reviewers. Any product that may be evaluated in this article, or claim that may be made by its manufacturer, is not guaranteed or endorsed by the publisher.
